# Origin of vertical orientation in two-dimensional metal halide perovskites and its effect on photovoltaic performance

**DOI:** 10.1038/s41467-018-03757-0

**Published:** 2018-04-06

**Authors:** Alexander Z. Chen, Michelle Shiu, Jennifer H. Ma, Matthew R. Alpert, Depei Zhang, Benjamin J. Foley, Detlef-M. Smilgies, Seung-Hun Lee, Joshua J. Choi

**Affiliations:** 1 Department of Chemical Engineering, University of Virginia, Charlottesville, VA 22904 USA; 2 Department of Physics, University of Virginia, Charlottesville, VA 22904 USA; 3 Cornell High Energy Synchrotron Source, Cornell University, Ithaca, NY 14853 USA

## Abstract

Thin films based on two-dimensional metal halide perovskites have achieved exceptional performance and stability in numerous optoelectronic device applications. Simple solution processing of the 2D perovskite provides opportunities for manufacturing devices at drastically lower cost compared to current commercial technologies. A key to high device performance is to align the 2D perovskite layers, during the solution processing, vertical to the electrodes to achieve efficient charge transport. However, it is yet to be understood how the counter-intuitive vertical orientations of 2D perovskite layers on substrates can be obtained. Here we report a formation mechanism of such vertically orientated 2D perovskite in which the nucleation and growth arise from the liquid–air interface. As a consequence, choice of substrates can be liberal from polymers to metal oxides depending on targeted application. We also demonstrate control over the degree of preferential orientation of the 2D perovskite layers and its drastic impact on device performance.

## Abstract (web summary)

It is desirable to align the two-dimensional perovskite layers vertical to the electrodes to maximize device performance but the formation mechanism is unclear. Here Chen et al. reveal that the film formation starts at the liquid-air interface and is thus independent of the choice of substrates.

## Introduction

Metal halide perovskites (MHPs) are poised to revolutionize the field of optoelectronic materials with their phenomenal performance advancement in solar cells^1–5^, light-emitting diodes (LEDs)^6–9^, photodetectors^10–14^, and lasers^15–17^. MHPs are unique in that they combine low-cost solution processability with superb electronic quality that is comparable to, or surpasses, that of the state-of-the-art epitaxial grown semiconductors^18–21^. Moreover, MHPs enable lightweight flexible device applications due to the fact that they can be deposited on various substrates at low temperature (<150 °C)^22–25^.

Despite their enormous potential, instability of MHPs is currently a major challenge to their device applications. Recently, two-dimensional (2D) Ruddlesden–Popper MHPs have been identified as materials that can potentially combine high-performance and long-term stability^26–31^. Going from three-dimensional (3D) perovskite *AMX*_3_ (*A* = CH_3_NH_3_^+^, HC(NH_2_)_2_^+^, Cs^+^, Rb^+^; *M* = Sn^2+^, Pb^2+^; *X* = Cl^−^, Br^−^, I^−^) to 2D *B*_2_*A*_*n*−1_*M*_*n*_*X*_3*n*+1_ (*B* = R-NH_2_^+^), bulky long-chain ammonium cations are introduced to confine layers of metal halide octahedra in two dimensions. 2D MHPs exhibit many of the superb optoelectronic properties of their 3D counterparts while providing an opportunity to tune the properties by controlling the thickness of the metal halide layers through the quantum confinement effect^32^. Due to the bulky long-chain surface terminating ligands that are hydrophobic, 2D MHPs show drastically improved stability against humidity compared to 3D MHPs^26–31^.

A critical requirement for high-performance 2D MHP optoelectronic devices is to align the 2D MHP layers perpendicular to the electrode layers. This is because the bulky ammonium cations that separate the 2D metal halide slabs are electrically insulating. For devices such as solar cells and LEDs, wherein the MHP thin films are sandwiched between electrodes, vertical alignment of 2D MHPs is desired to obtain efficient charge transport to the electrodes^27, 28^. However, the self-assembly processes through which 2D MHPs can be aligned vertically remain a mystery and cannot yet be controlled.

Here we reveal a mechanism responsible for the formation of vertically orientated 2D MHPs in thin films using grazing incidence wide angle X-ray scattering (GIWAXS). The thin films of BA_2_MA_3_Pb_4_I_13_ (BA = butylammonium, MA = methylammonium) are fabricated on various substrates using a simple solution processing at a fixed low temperature between 25 and 140 °C. Our ex situ GIWAXS results show different degrees of preferential vertical orientation depending on the processing conditions. Solar cell performance measurements show that BA_2_MA_3_Pb_4_I_13_ with a higher degree of vertical orientation results in drastically higher power conversion efficiency than those with more random orientations. In order to understand how the vertical orientation forms, we have performed systematically designed in situ GIWAXS experiments. Most importantly, our results show that heterogeneous nucleation and growth of BA_2_MA_3_Pb_4_I_13_ occur at the liquid–air interface to form a top-crust with strong vertical orientation. We confirm this by repeating the GIWAXS measurements before and after selectively removing the top crust. The formation of vertically orientated 2D MHPs at the anisotropic environment of the liquid–air interface is likely due to preference for the aliphatic chain of butylammonium molecules bound to the metal halide slabs to remain in the solution environment. Knowing the origin of the preferential orientation allows us to rationally tune the degree of vertical orientation of 2D MHP with similar thin film morphology. As a consequence of the top-down growth mechanism, high-quality 2D MHP thin films can be deposited on various substrates ranging from polymers to metal oxides.

## Results

### Structural and optical characterization

BA_2_MA_3_Pb_4_I_13_ films were synthesized using a simple method of sequential spin-coating and thermal annealing steps (see Methods section for details). The precursor solution was prepared by dissolving stoichiometric amounts of lead iodide (PbI_2_), methylammonium iodide (MAI), and butylammonium iodide (BAI) in dimethylacetamide (DMAc) solvent. DMAc was chosen because it suppresses formation of intercalated intermediate structures leading to smooth morphology^33^. The solution was spin-coated onto a substrate briefly and, before the liquid film dried and turned dark, the sample was transferred to a hotplate set at various temperatures ranging from 25 to 140 °C (Supplementary Fig. 2). The MHP nucleation and growth then occur on the hotplate and result in a dark colored thin film. Our DMAc spin-coating method yields a uniform and reflective surface with a complete coverage, as evidenced by the scanning electron microscope (SEM) image shown in Supplementary Fig. 1c. The XRD pattern from the BA_2_MA_3_Pb_4_I_13_ film indicates an orthorhombic (101) vertical crystallographic orientation with respect to the substrate^27, 28^ as shown in Supplementary Fig. 3b. The bandgap energy was measured to be 1.67 eV using absorbance and photoluminescence spectroscopy (Supplementary Fig. 1b and 4) which is consistent with the previous literature^28, 34^. Excitonic peaks at locations consistent with lower layer (*n* < 4) impurities are also present in absorption spectrum (Supplementary Fig. 1b), as typically observed in solution processed BA_2_MA_3_Pb_4_I_13_ thin films^8, 9, 35, 36^. For comparison with our DMAc method, we have also fabricated BA_2_MA_3_Pb_4_I_13_ thin films using previously reported methods such as dimethylformamide (DMF)-based spin-coating^27^ and hot casting^28^. In the “hot-casting” process, precursor solution with DMF as a solvent is casted on a hot spinning substrate to crystalize rapidly^28^. Detailed fabrication procedures and characterization results of these films are described in Methods section and Supplementary Fig. 3 and 5, respectively.

**Fig. 1:**
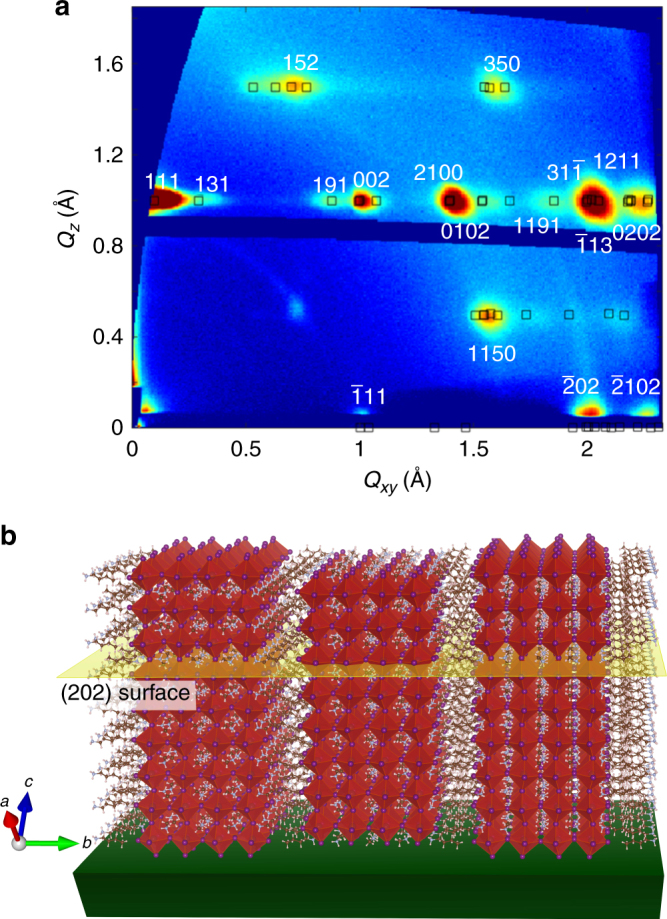
GIWAXS pattern with indexing and illustration of the crystallographic orientation. **a** GIWAXS pattern of a BA_2_MA_3_Pb_4_I_13_ thin film spin-coated from DMAc solution. The experimental peak positions agree well with simulation (black square) from an orthorhombic (101) vertically oriented BA_2_MA_3_Pb_4_I_13_ structure. **b** Illustration of an orthorhombic (101) vertically oriented 2D perovskite structure, with (202) planes parallel to the substrate

To obtain more in-depth structural information on the BA_2_MA_3_Pb_4_I_13_ thin films, grazing incidence wide angle X-ray scattering (GIWAXS) was performed on our samples^37–39^ (see Methods section for details). In the 2D GIWAXS patterns, diffraction intensity concentrated in spots indicates preferential crystallographic orientation with respect to the substrate while, in contrast, the diffraction intensity spread out into rings indicates randomly oriented crystals^37, 38^. A GIWAXS pattern from a BA_2_MA_3_Pb_4_I_13_ thin film prepared with our DMAc method is shown in Fig. 1a. The diffraction intensity is highly concentrated in spots, indicating a high degree of preferential crystallographic orientation. The pattern can be well indexed with a calculated pattern from the structure of BA_2_MA_3_Pb_4_I_13_^40^ with (202) planes parallel to the substrate. Line-cuts of the GIWAXS pattern are consistent with the calculated pattern as shown in Supplementary Fig. 6. The crystallographic structure determined by our GIWAXS results is illustrated in Fig. 1b that shows vertically oriented BA_2_MA_3_Pb_4_I_13_.

### Nucleation and growth mechanism of vertical orientation

It is intriguing how the strong vertical orientation can form from solution through the spin-coating-annealing process. Homogeneous nucleation within the bulk of the solution results in growth of randomly oriented crystals due to the isotropic environment^41, 42^. In certain cases, crystals from homogeneous nucleation can result in a preferential orientation upon deposition on a substrate if the crystal has anisotropic dimensions and has a preferential orientation that maximizes interactions between the crystals and the substrate, such as van der Waals (vdW) attraction. However, such a case typically induces horizontal, rather than vertical, orientation of the 2D crystal plates to maximize the vdW attraction^43^. Therefore, the observed strong vertical orientation in our films suggest that homogeneous nucleation is suppressed and, instead, heterogeneous nucleation at an interface is dominant. The question that arises is at which interface the heterogeneous nucleation occurs, the substrate–liquid interface, liquid–air interface or both? Finding the answer to this question is crucial in understanding the formation mechanism of vertically oriented the 2D MHP thin films that are required for high performance devices.

**Fig. 2:**
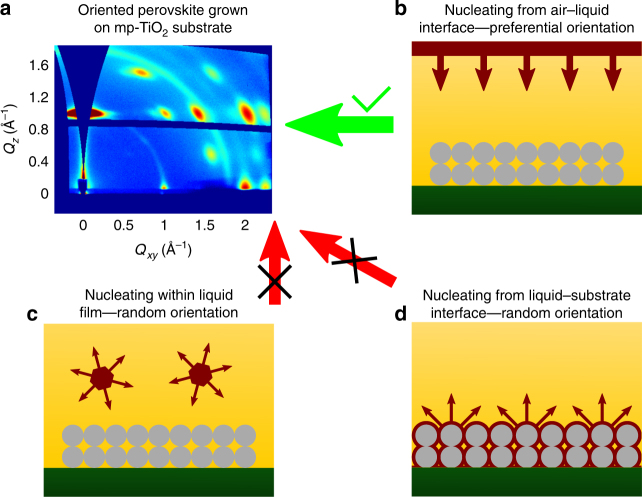
Possible scenarios for nucleation with mesoporous TiO_2_ substrates. **a** GIWAXS pattern of BA_2_MA_3_Pb_4_I_13_ thin film formed on mesoporous TiO_2_ (mp-TiO_2_) substrates indicates a strong vertical orientation. The continuous ring at 1.8 Å is diffraction signal from mp-TiO_2_ substrate. **b**–**d** Illustration of possible crystallization processes from three different nucleation sites: liquid–air interface **b**, within bulk liquid **c**, and substrate–liquid interface **d**. The gray circle stacks represent mp-TiO_2_ substrate, the brown species represent BA_2_MA_3_Pb_4_I_13_ and the brown arrows represent the crystallization direction. With the mp-TiO_2_ substrate, only the nucleation and growth from the liquid–air interface scenario is consistent with formation of a vertically oriented BA_2_MA_3_Pb_4_I_13_ thin film

First, to check if the nucleation occurs at the substrate–liquid interface, we self-assembled BA_2_MA_3_Pb_4_I_13_ thin films using our DMAc method on various different substrates including PEDOT, SnO_2_, NiO_*x*_, TiO_*x*_, and mesoporous TiO_2_. We found that strong vertical orientation of BA_2_MA_3_Pb_4_I_13_ on all of these substrates (Supplementary Fig. 7). It is particularly striking that highly oriented BA_2_MA_3_Pb_4_I_13_ is obtained even on mesoporous TiO_2_ substrate. If nucleation and subsequent growth happens at the liquid–substrate interface, we would expect the highly tortuous and rough surface of mesoporous TiO_2_ substrate to result in crystal growth in all directions (Fig. 2d), resulting in mostly random orientation of 2D MHP crystals. However, even on a mesoporous TiO_2_ substrate, the GIWAXS pattern shows a strong vertical orientation in the BA_2_MA_3_Pb_4_I_13_ thin film (Fig. 2a). The ring at *q* around 1.8 Å^−1^ corresponds to diffraction intensity from the mesoporous TiO_2_ substrate at the bottom (Supplementary Fig. 8), indicating that the X-ray beam is probing all the way to the substrate surface. The lack of dependence on different substrates as well as the formation of strong vertical orientation regardless of the tortuous and uneven surface of the mesoporous TiO_2_ substrate indicates that the nucleation does not occur at the substrate–liquid interface. As discussed previously, nucleation and growth within the liquid bulk (Fig. 2c) are also expected to be absent as such a case would result in randomly oriented crystals due to the isotropic environment in the bulk solution, or horizontally oriented crystals upon deposition on the substrate. Based on these results, the most likely scenario is that the vertically oriented BA_2_MA_3_Pb_4_I_13_ crystals originate from the anisotropic environment of liquid–air interface, regardless of the substrate choice as illustrated in Fig. 2b.

**Fig. 3:**
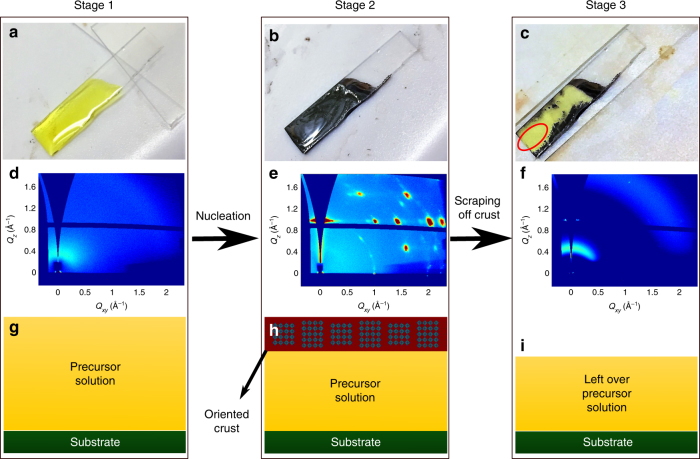
Optical images and GIWAXS patterns and illustrations of top-crust scraping test. In stage 1, the precursor solution is deposited on a glass substrate. Optical image **a** and GIWAXS pattern **d** shows yellow liquid with no crystalline species **g**. In stage 2, immediately after the surface turns dark upon thermal annealing **b**, GIWAXS pattern **e** shows a vertically oriented BA_2_MA_3_Pb_4_I_13_ top-crust **h**. The top-crust is removed by a blade-scraping setup in stage 3 to expose the yellow precursor solution underneath that has not yet crystallized **c**. GIWAXS pattern of the exposed spot **f** shows diffuse scattering only from its non-crystalline left-over precursor solution **i**

Next, we have performed in situ GIWAXS experiments to check for the nucleation at the liquid–air interface. As illustrated in Fig. 2b, nucleation starting from the liquid–air interface can form a top-crust of highly oriented solid crystal film as a template for further crystal growth, with non-crystalline precursor solution underneath. To confirm this scenario, we designed a home-made blade setup (Supplementary Fig. 9) to selectively remove the top-crust during the self-assembly process in the following way. The precursor solution was deposited on a glass substrate (stage 1). The wet film was then annealed at 60 °C. Shortly after the whole film turned black (stage 2), the top-crust of the film was scraped off with a sharp blade (stage 3). The blade was configured to suspend above the substrate so that it did not touch the substrate surface during the process. GIWAXS patterns were taken at each stage to probe the presence of BA_2_MA_3_Pb_4_I_13_. In stage 1, the deposited precursor is a yellow liquid that has not yet crystallized (Fig. 3a), and the GIWAXS pattern shows no crystalline diffraction peaks in the wet film (Fig. 3d) and only shows diffuse scattering from the solution. The precursor liquid film was then annealed at 60 °C. Immediately after the film surface turned dark (Fig. 3b), in stage 2, the GIWAXS pattern shown in Fig. 3e indicates the formation of a vertically oriented crystalline BA_2_MA_3_Pb_4_I_13_ film. However, when viewing from the other side of the glass substrate, liquid solution was observed (Supplementary Fig. 10), indicating that the precursor solution underneath the oriented perovskite crust has not yet crystallized. In stage 3, the perovskite top-crust was scraped off with a blade. The scraping of the top-crust exposed the yellow precursor solution underneath (Fig. 3c). GIWAXS measurement on the scraped spot (Fig. 3f) showed diffuse scattering from the liquid only with no crystalline peaks, confirming the visual observation. The fact that the diffuse scattering ring narrows and shifts to higher *q* suggests that there could be changes in structure of precursors, but still without any long-range periodicity.

**Fig. 4:**
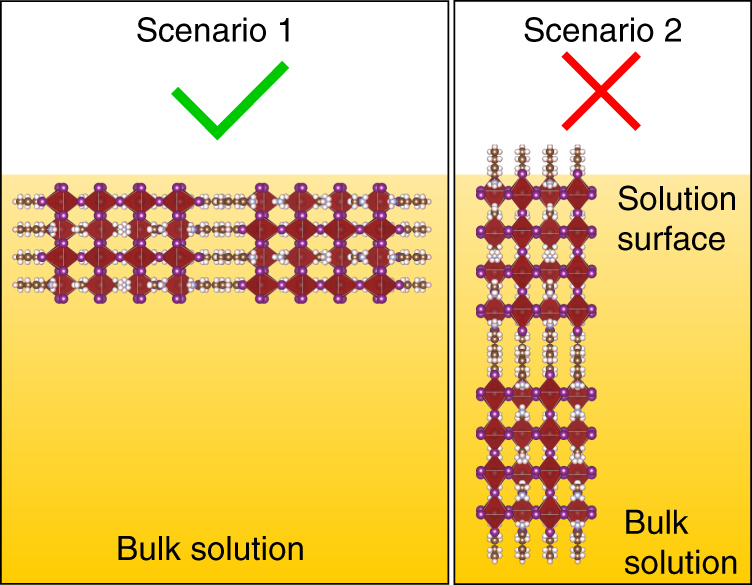
Schematics of preferred orientation at liquid–air interface. The two possible nucleus orientations at the anisotropic precursor solution–air interface are shown in scenario 1 and scenario 2. The vertical orientation in crystallized crust confirmed by GIWAXS indicates that scenario 1 is occurring

This test confirms that nucleation of BA_2_MA_3_Pb_4_I_13_ occurs at the liquid–air interface. It has recently been reported that surface tension makes nucleation and growth at the liquid–air interface of perovskite precursor solution more favorable^44^. A similar process may be responsible for the 2D perovskite system as well. The confirmation of the crystallization at the liquid–air interface sheds light on the origin of the peculiar preferential vertical orientations. The orientation of the nucleus formed at the liquid–air interface and subsequent crystal growth determine the crystallographic orientation. Our observation of the vertically oriented BA_2_MA_3_Pb_4_I_13_ thin film crystallizing from the liquid–air interface indicates that the initial nuclei are oriented in vertical configuration as illustrated in Fig. 4a, rather than the horizontal configuration as in Fig. 4b. It is possible that, with the butylammonium molecules bound to the lead iodide slabs such that the butyl chain are pointing toward outside, vertical orientation of the 2D perovskite nuclei is favored such that the butyl chains stay well inside the solution (Fig. 4a). The formation of vertically oriented BA_2_MA_3_Pb_4_I_13_ also indicates that a monolayer of butylammonium molecules as typically seen in Langmuir–Blodgett films^45^ with butyl chains vertical to the liquid–air interface, likely does not form in this situation. If such a monolayer of butylammonium molecules formed at the liquid–air interface, it would have induced horizontal orientation of BA_2_MA_3_Pb_4_I_13_. However, our observation of vertically oriented BA_2_MA_3_Pb_4_I_13_ suggests that is not the case. It is likely that the butylammonium molecules are not able to form a monolayer at the liquid–air interface due to either DMAc–butylammonium–air interaction does not favor formation of monolayer thin film, or a majority of butylammonium complexing with lead iodide species.

### Controlling degree of vertical orientation

**Fig. 5:**
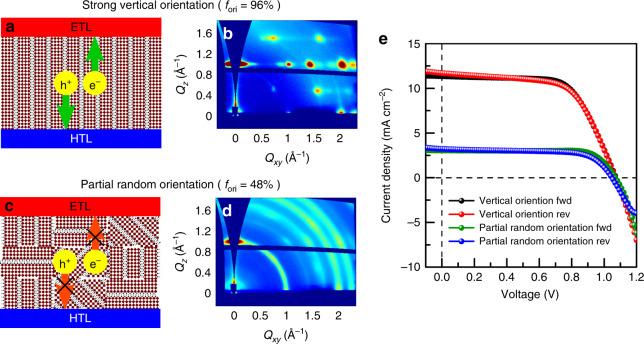
Charge transport and device performance with different degrees of orientation. Pre-crystallization annealed film with strong vertical orientation indicated by GIWAXS in **b** provides direct pathway for electron and hole extraction in **a**, while in post-crystallization annealed film with partial random orientation in **d**, charge carriers need to hop through electrically insulating organic ligands to reach electron transporting layer (ETL) and hole transporting layer (HTL), which hinders charge extraction in **c.** The percentage of oriented crystallites in the perovskite film is defined as *f*_ori_. Performance of solar cell with pre-crystallization annealing method (red and black lines) is significantly higher than that of post-crystallization annealing method (green and blue lines) in **e**

Knowing the origin of the preferential orientation allows us to rationally control the degree of orientation by tuning the crystallization conditions. We refer to the crystallization process described so far as “pre-crystallization annealing” where the BA_2_MA_3_Pb_4_I_13_ film crystallizes on a hotplate at elevated temperature with minimal spin-induced convection. In contrast, if the precursor solution film is left for a longer period during spin-coating process such that the film gets dried while spinning, the evaporation rate of the solvent can be kept high with spin-induced convection while the lack of heating keeps the diffusion rates of precursor species low. This can induce a significant amount of homogeneous nucleation throughout the solution which will result in more randomly oriented BA_2_MA_3_Pb_4_I_13_. The thermal annealing is then applied after the crystallization has been completed during spinning. We call this procedure as “post-crystallization annealing” method. Figure 5b, d shows that the films made using the two different methods show very different degrees of the preferential vertical orientation. Figure 5b shows that the pre-crystallization annealed film shows a degree of preferential vertical orientation, *f*_ori_, of 96%. Fig 5d, on the other hand, shows that the post-crystallization annealed film has *f*_ori_ of 48%, which means that about half of the BA_2_MA_3_Pb_4_I_13_ plates are randomly oriented. The details regarding quantification of *f*_ori_ is available in Method section, Supplementary Fig. 11 and Supplementary Table 1. Although they are fabricated from the same precursor, substrate, spin speed, and glovebox environment, with similar film morphology (Supplementary Fig. 12), different thermal annealing timing results in drastically different degree of preferential orientation.

### Impact of orientation on solar cell performance

The control over the degree of preferential orientation in BA_2_MA_3_Pb_4_I_13_ thin films enabled us to systematically compare its effect on solar cell performance. It is expected that a vertically oriented 2D perovskite thin film provides direct pathway for electron and hole transport, while the charge transport is hindered by hopping across the long-chain organic surface ligands in a more randomly oriented film (Fig. 5a, c). To test this, we fabricated and characterized BA_2_MA_3_Pb_4_I_13_ solar cells using the pre- and post-crystallization annealing methods. The structure of solar cells was ITO/PEDOT:PSS/BA_2_MA_3_Pb_4_I_13_/PCBM/Cu (see Methods section for detailed procedure), and their performance is shown in Fig. 5e and Table 1. The device performance statistics based on 30 devices are shown in Supplementary Fig. 13. The *J–V* curve of BA_2_MA_3_Pb_4_I_13_ solar cells made with both methods show a slight hysteresis behavior, which is frequently observed in 3D perovskite solar cells, indicating mechanisms causing hysteresis in 3D perovskite solar cells also exist in 2D perovskite, including capacitive effect, trapping and detrapping of carriers, ion migration and ferroelectric effect^46^.

**Table 1:** Solar cell parameters of the pre- and post-crystallization annealed devices

Method	Scanning direction	*V*_oc_ (V)	*J*_sc_ (mA cm^−2^)	FF	*R*_series_ (Ω cm^2^)	*R*_shunt_ (Ω cm^2^)	Efficiency (%)
Pre-crystallization annealing	Fwd	1.07	11.3	64.8	22.3	1653	7.9
	Rev	1.07	11.7	61.8	21.7	594	7.7
Post-crystallization annealing	Fwd	1.07	3.0	78.7	36.0	7800	2.6
	Rev	1.05	3.2	67.6	38.7	1062	2.3

The pre-crystallization annealed film with a strong vertical orientation outperforms the films with partial random orientation due to a significantly higher short circuit current density (*J*_sc_) and lower series resistance (*R*_s_), while both devices have similar open circuit voltage (*V*_oc_). Both films have similar thicknesses of around 325 nm, absorbance spectra and optical density (Supplementary Fig. 14 and 15). Therefore, the higher *J*_sc_ of the pre-crystallization annealed solar cell is unlikely due to different amounts of light absorption. Shunt resistance (*R*_sh_) from both set of devices have high values (Table 1), indicating the devices are not significantly affected by shunting from rough morphology and incomplete coverage. Combined together, these observations are consistent with the expectation of superior charge transport properties with vertical crystallographic orientation compared to randomly oriented 2D MHPs, which results in improved *J*_sc_. Our results clearly show that a strong vertical orientation is necessary for achieving high performance in optoelectronic devices.

## Discussion

In summary, we reveal that vertically oriented 2D MHP BA_2_MA_3_Pb_4_I_13_ nucleates and grows from the precursor liquid–air interface. By comparing electronic properties of vertically oriented and partially randomly oriented thin films, we find that a strong vertical orientation is necessary for optimal solar cell performance. This insight on the 2D MHP self-assembly and its impact on optoelectronic device performance will accelerate the progress toward a wide-spread application of 2D MHPs devices.

## Methods

### Materials and instruments

PEDOT:PSS Al 4083 (Clevios) was filtered with a 0.45 µm size filter before use. Methylammonium iodide (MAI) and butylammonium iodide (BAI) were purchased from Dyesol. PbI_2_ (99.9985%) was purchased from Alfa Aesar. Anhydrous dimethylacetamide (DMAc) (99.8%), anhydrous DMF (99.8%) and bathocuproine (BCP) (99.99%) were purchased from Sigma Aldrich. Indium tin oxide (ITO) substrates (15 Ω cm^−2^) were purchased from Kintec. Phenyl-C61-butyric acid methyl ester (PCBM) (99.5%) was purchased from Nano-C. Copper pellets were purchase from Kurt Lesker.

### X-ray diffraction

XRD was performed on a PANalytical X’pert system at 40 kV and 40 mA.

### GIWAXS measurements and indexing method

GIWAXS was performed at D-1 beamline at Cornell High Energy Synchrotron Source (CHESS) using X-rays with a wavelength of 1.162 Å, a custom precision goniometer, and a Pilatus 200k 2D pixel array detector (Dectris). The temperature of the custom-built sample holder was controlled by a temperature controller (Digi-Sense) and the temperature was monitored during X-ray data collection. A typical X-ray incidence angle was 0.7°. The data reduction^47^ and calculation of the location and intensity of the diffraction peaks with PowderCell were performed using a previously reported structure^40^.

### Perovskite film fabrication

Pre-crystallization annealing method: 0.75 M BA_2_MA_3_Pb_4_I_13_ solution in DMAc was made by dissolving 0.75 M PbI_2_, 0.5625 M MAI and 0.375 M BAI with stirring. The solution was filtered with a 0.22 µm filter and then deposited on the substrate by spinning at 2000 r.p.m. After about 20 s of spin-coating the slide was taken off the spincoater and immediately placed on a hotplate to anneal at 120 °C for 10 min, where the precursor crystallized and turned dark brown. Post-crystallization annealing method: 0.75 M BA_2_MA_3_Pb_4_I_13_ solution dissolved in DMAc was filtered with a 0.22 µm filter and then deposited on the substrate by spinning at 2000 r.p.m. After 60 s of spin-coating the slide had already dried and turned dark brown, and was taken off the spincoater and placed on a hotplate to anneal at 120 °C for 10 min.

### Device fabrication and characterization

ITO slides were cleaned by sequentially sonication in soap water, deionized water and ethanol, then treated with ozone plasma for 10 min before use. PEDOT:PSS was deposited by spin-coating at 6000 r.p.m. for 60 s, followed by annealing at 120 °C for 15 min. The slides were then transferred into a N_2_ filled glovebox. Pre-and-post-crystallization annealing methods were carried out to form the perovskite active layers, as described above. A layer of PCBM was deposited by spin-coating a freshly filtered 10 mg ml^−1^ solution in chloroform at 3000 r.p.m. for 60 s, followed by annealing at 100 °C for 10 min. After the slides cooled down, a layer of BCP was spin-coated at 4000 r.p.m. with a 0.5 mg ml^−1^ solution in isopropanol. 50 nm Cu electrode was then thermally evaporated in ultra-high vacuum (<10^−5^ torr). Device active area is 0.03 cm^2^, defined by the overlap between ITO substrate and metal electrode, as well as an optical mask. The devices were tested under simulated Air Mass 1.5 irradiance of 100 mW cm^−2^ light source calibrated by a reference Si solar cell.

### Calculating degree of preferential orientation

Bragg peaks [2, 0, 0] and [0, 0, 2] are fit with Gaussian distribution and the fitting results and coefficients are in Supplementary Fig. 13 and Supplementary Table 1. $\Delta Q_{{\mathrm{rad}}} = \Delta Q$ and $\Delta Q_{{\mathrm{tan}}} = Q_{\mathrm{c}} \times \Delta \chi$ are the fitted FWHMs in radial and tangential directions. The orientation correlation length $\xi _{{\mathrm{tan}}}$ is related to the tangential width of the Bragg peaks and can be calculated with the Scherrer equation, $\xi _{{\mathrm{tan}}} = \frac{{2\pi \times 0.9}}{{\sqrt {\left( {\Delta Q_{{\mathrm{tan}}}} \right)^2 - \left( {\Delta Q_{{\mathrm{res}}}} \right)^2} }}$. The ratio of orientated crystallites, $f_{{\mathrm{ori}}} = \frac{{A_{{\mathrm{ori}}}}}{{A_{{\mathrm{ori}}} + A_{{\mathrm{iso}}}}}$, where $A_{{\mathrm{ori}}}$ ($A_{{\mathrm{iso}}}$) is the amount of orientated (isotropic) crystallites, is calculated with the area under the fitted Gaussian peak (without the flat line) divided by the total area under $\widetilde I\left( \chi \right)$ in Supplementary Fig. 11b^48^.”

### Data availability

The data that support the findings of this study are available from the corresponding author upon request.

## Electronic supplementary material

Supplementary Information
