# Supplementary material for: Origin of vertical orientation in two-dimensional metal halide perovskites and its effect on photovoltaic performance
Source: Nat Commun. 2018 Apr 6;9:1336. doi: 10.1038/s41467-018-03757-0 (PMC5889398; doi:10.1038/s41467-018-03757-0)
Supplement: Supplementary file 1 — Supplementary Information [file 41467_2018_3757_MOESM1_ESM.pdf]

SUPPLEMENTARY INFORMATION

Origin of vertical orientation in two-dimensional metal halide perovskites and its effect on photovoltaic performance

Alexander Z. Chen[1], Michelle Shiu[1], Jennifer H. Ma[1], Matthew R. Alpert[1], Depei Zhang[2], Benjamin J. Foley[1], Detlef-M. Smilgies[3], Seung-Hun Lee[2] and Joshua J. Choi[1,*]

[1]Department of Chemical Engineering, University of Virginia, Charlottesville, Virginia 22904, USA

[2]Department of Physics, University of Virginia, Charlottesville, Virginia 22904, USA.

[3]Cornell High Energy Synchrotron Source, Cornell University, Ithaca, New York 14853, USA.

*Corresponding Author. E-mail: jjc6z@virginia.edu

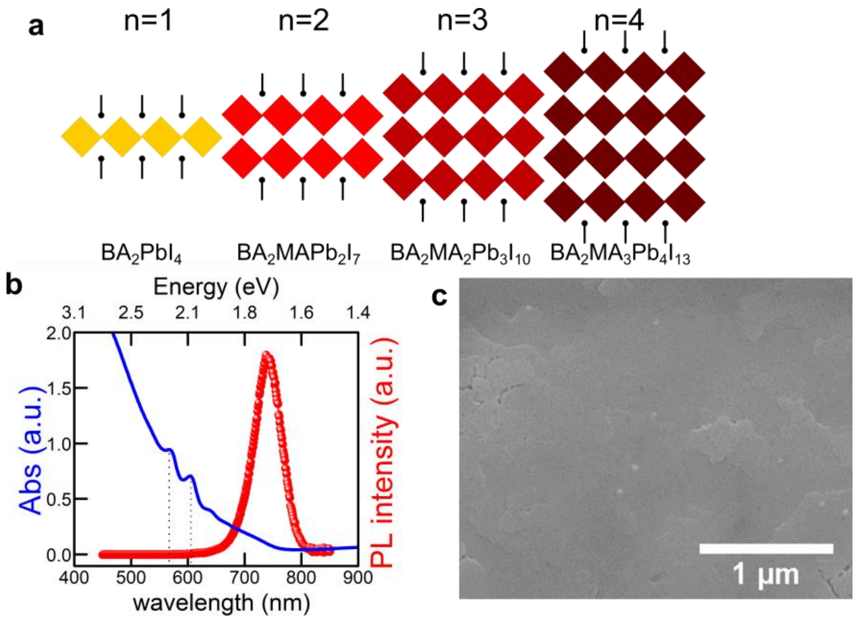

Supplementary Figure 1. Characterization of two dimensional perovskite thin films. **a**. An illustration of the structure of 2D perovskite with number of layers (n) ranging from 1 - 4. Each colored diamond represents a Pb-I octahedron, and the black round arrows represent the butylammonium surface ligands. **b**. Absorbance and photoluminescence spectra of a 4 layer $BA_2MA_3Pb_4I_{13}$ thin film made by DMAc method. **c**. SEM image of a $BA_2MA_3Pb_4I_{13}$ thin film.

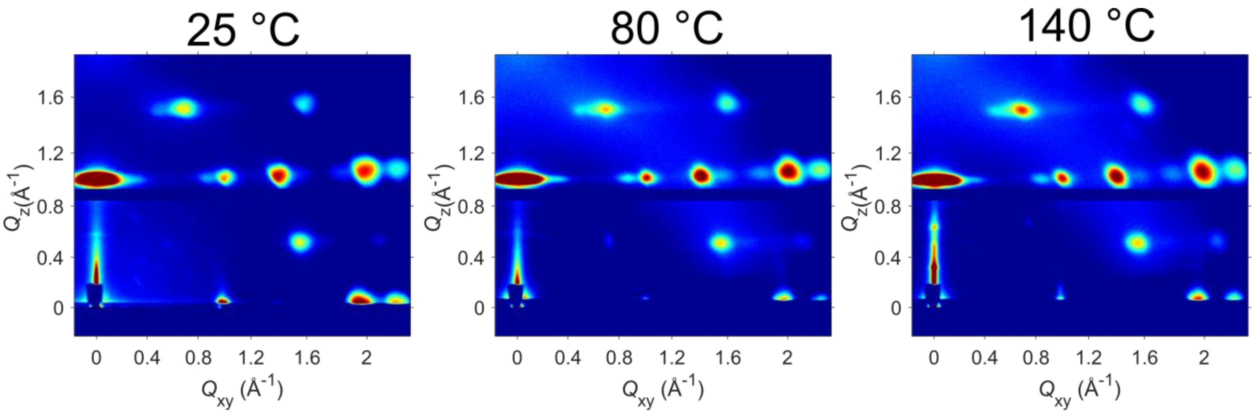

Supplementary Figure 2. GIWAXS patterns of $BA_2MA_3Pb_4I_{13}$ films with different annealing temperature from 25 °C to 140 °C. Even at room temperature, $BA_2MA_3Pb_4I_{13}$ crystallizes into a strong vertically oriented thin film using DMAc method. The weak small angle peak at Q_z = 0.63 Å^{-1} shown in the pattern from 140 ^{o}C sample could be due to lower layer (n < 4) impurities.

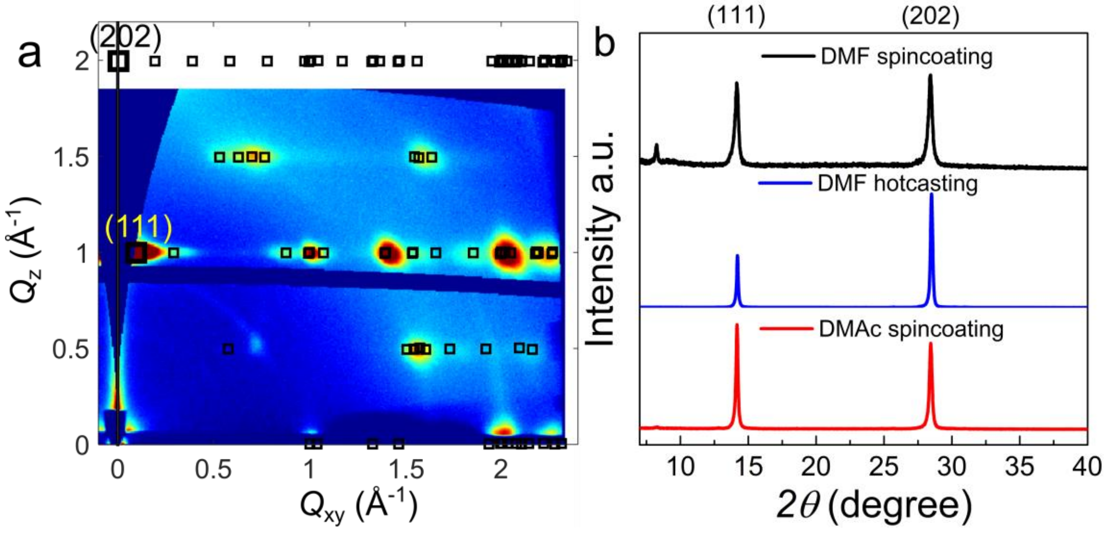

Supplementary Figure 3. Diffraction pattern from GIWAXS of $BA_2MA_3Pb_4I_{13}$ thin film from DMAc method (a) and XRD patterns of $BA_2MA_3Pb_4I_{13}$ thin film from DMF spin-coating, DMF hotcasting and DMAc methods (b). The XRD patterns indicate an orthorhombic (101) vertical crystallographic orientation of $BA_2MA_3Pb_4I_{13}$ thin films with respect to the substrate. The thin film from DMF spin-coating method has a diffraction peak at 8.25 degrees, which could be from either a solvent intercalation structure with long range order[1,2] or horizontally oriented components[3]. The different ratio in XRD peak intensity from films based on our DMAc method and DMF hotcasting method is due to the fact that, in one-dimensional XRD, the detector only probes diffraction peaks in the $Q_{xy} = 0$ line. Since peak (111) slightly deviates the $Q_{xy}=0$ line, XRD only detects signals from the peak broadening of (111), instead of directly probing the peak maximum. In comparison, peak (202) is on the $Q_{xy}=0$ line (out of range in the GIWAXS pattern) and can be directed collected by pXRD. So the peak intensity ratio of (111) and (202) highly depends on the peak broadening, and is not directly comparable between different samples using conventional XRD patterns only. The small angle peaks at $Q_z = 0$ Å^{-1} are not observed in the GIWAXS pattern. We hypothesize that this is due to either lack of long-range order in assemblies of 2D MHP plates or the imperfectly flat sample surface blocking the diffraction signals at $Q_z = 0$ Å^{-1} from being collected.

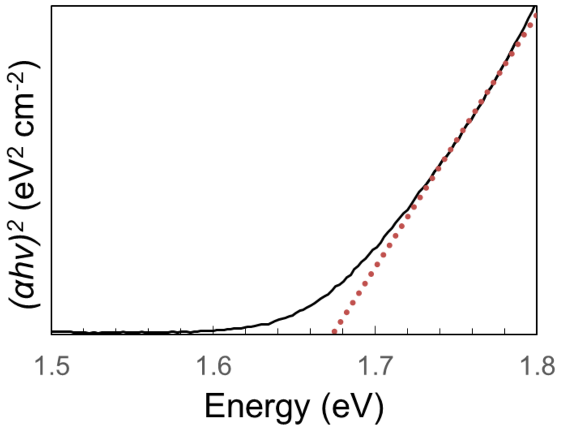

Supplementary Figure 4. Tauc plot of $BA_2MA_3Pb_4I_{13}$ thin film. The bandgap is determined to be 1.67 eV.

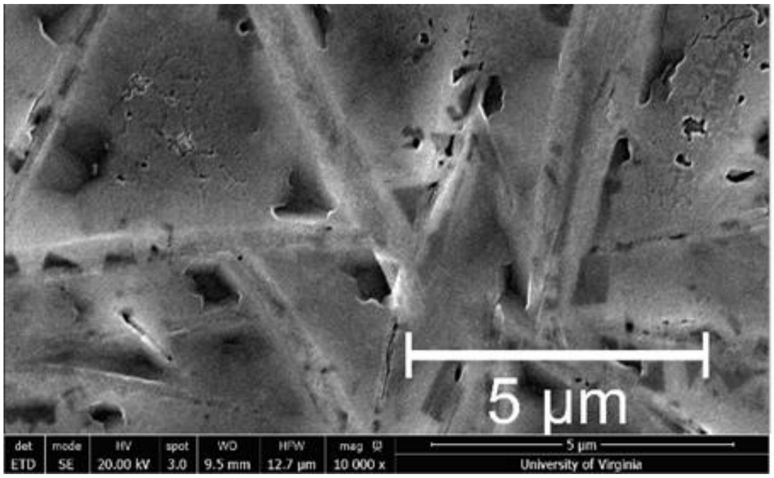

Supplementary Figure 5. SEM image of $BA_2MA_3Pb_4I_{13}$ thin film morphology from DMF spin-coating method. Unlike our DMAc method, DMF spin-coating method results in a thin film that is visibly rough, with an incomplete substrate coverage and uneven morphology.

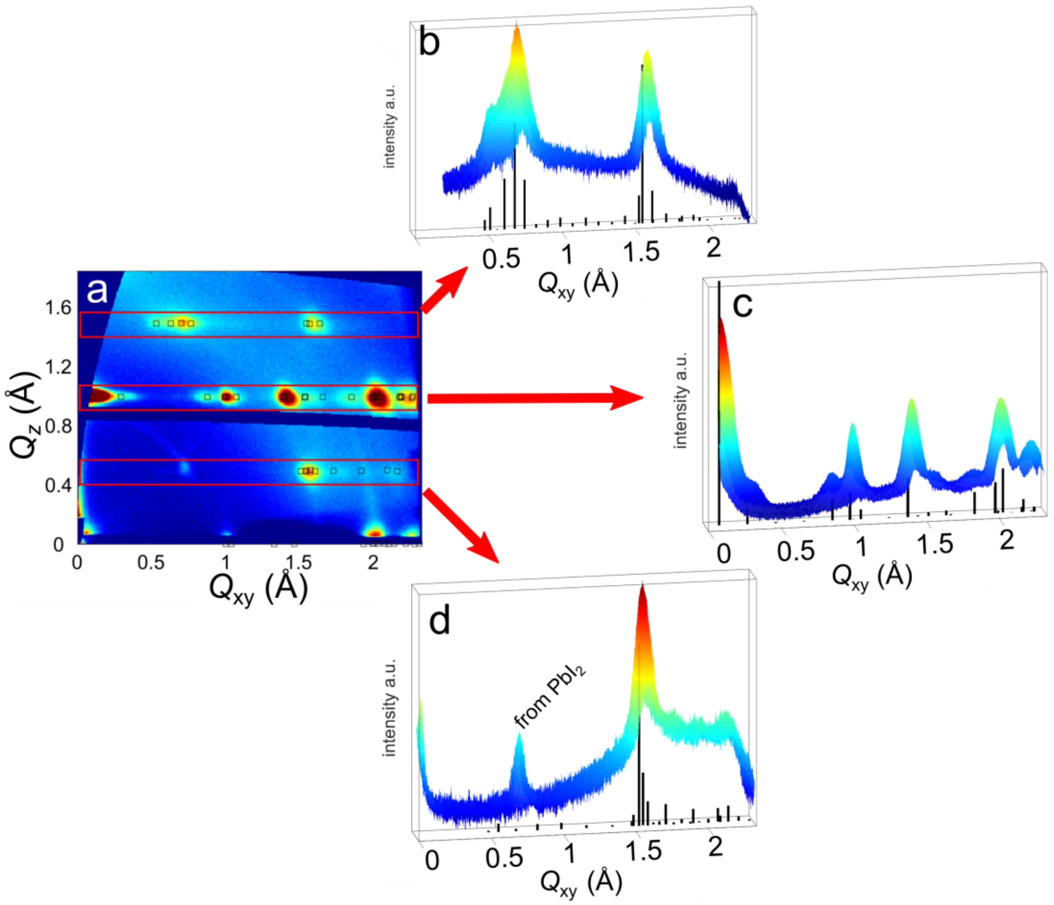

Supplementary Figure 6. A comparison between GIWAXS measurement and diffraction simulation. The linecuts are separated into three areas as marked on the GIWAXS pattern (a) and shown in (b, c, d), where the 3D surfaces represent experimental data and the bars are calculated by simulation. The unindexed peak in (d) are from PbI_2, as labelled in figure.

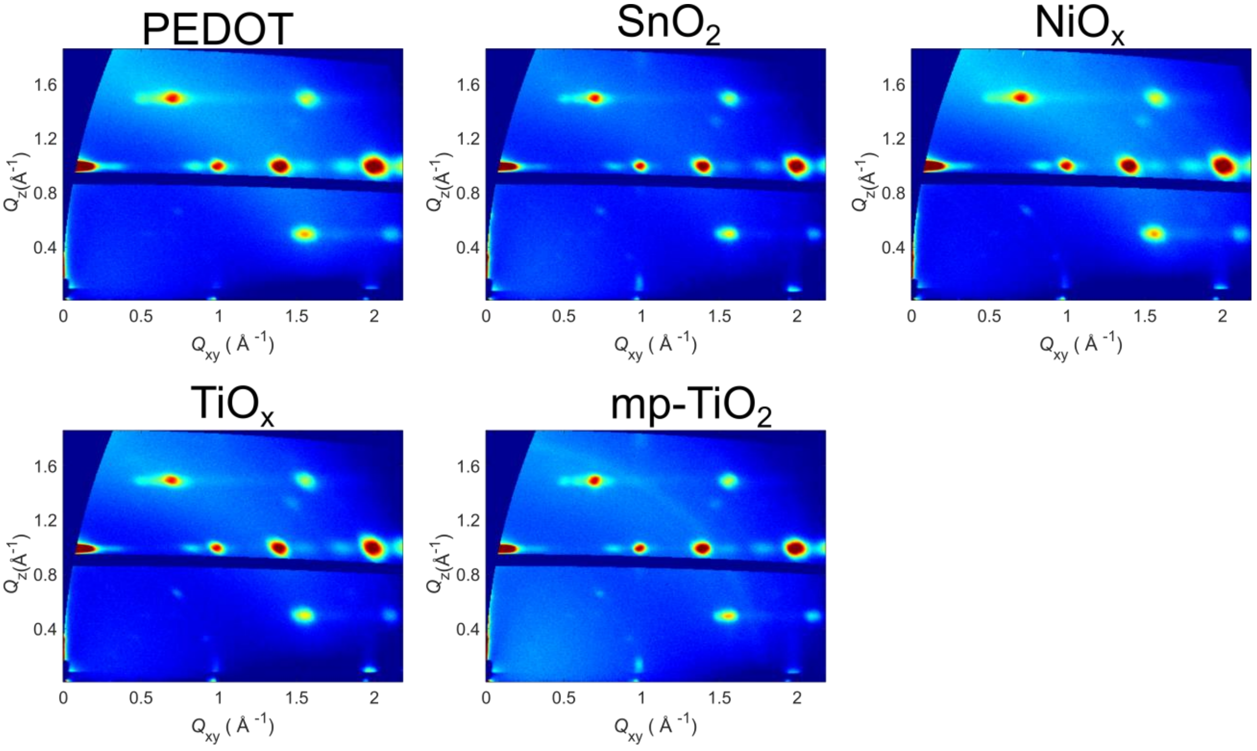

Supplementary Figure 7. Substrate dependence study shows strong vertical orientation occurs in all substrates tested, including PEDOT, SnO_2, NiO_x, planar TiO_x and mesoporous TiO_2.

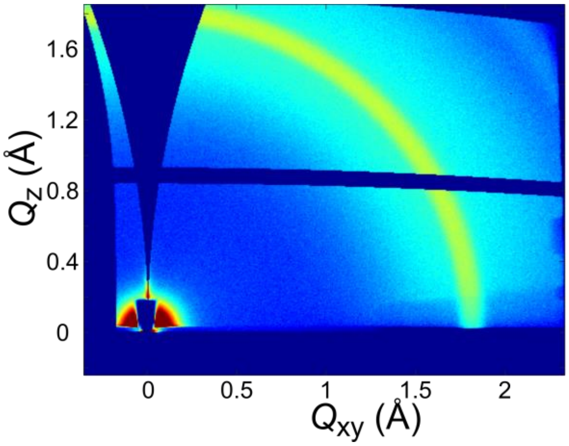

Supplementary Figure 8. GIWAXS pattern of a bare mesoporous TiO_2 substrate.

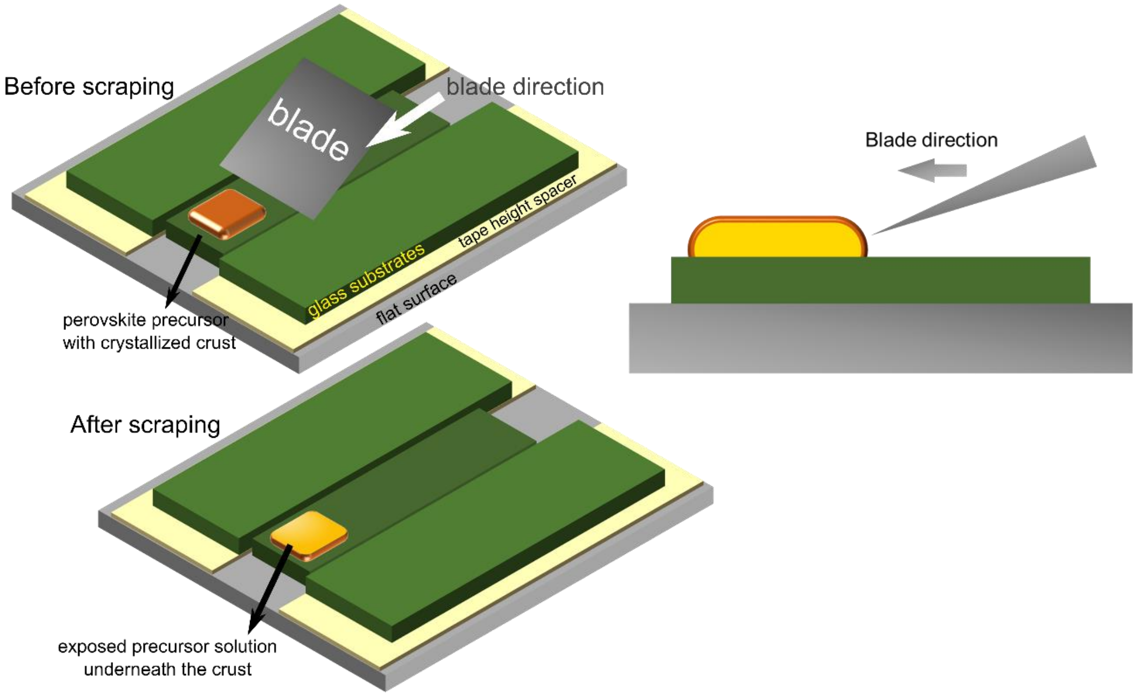

Supplementary Figure 9. Schematics of top-crust scraping test. Two glass substrates are placed on a flat clean surface, with scotch tape at the bottom as height spacer to elevate the blade. A glass substrate with the same thickness with a puddle of precursor deposited on top are briefly heat treated and turned dark, and then placed between the two elevated glass slides. A blade was then used to scrape off the crust of the puddle to reveal the yellow precursor underneath.

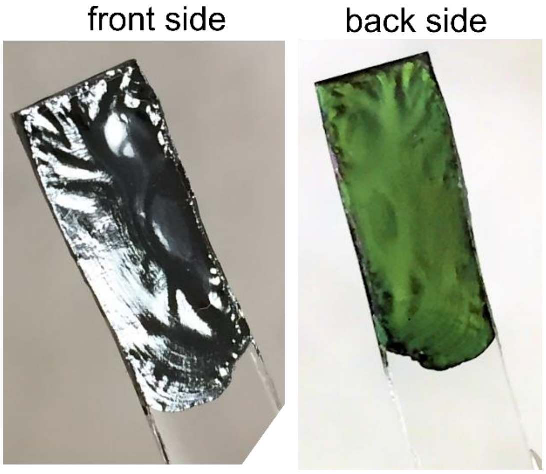

Supplementary Figure 10. Front and back side of a puddle of precursor solution immediately after a brief heat treatment. The front side shows a crystallized film with full perovskite coverage, while the liquid solution is visible from the back side underneath the perovskite crust.

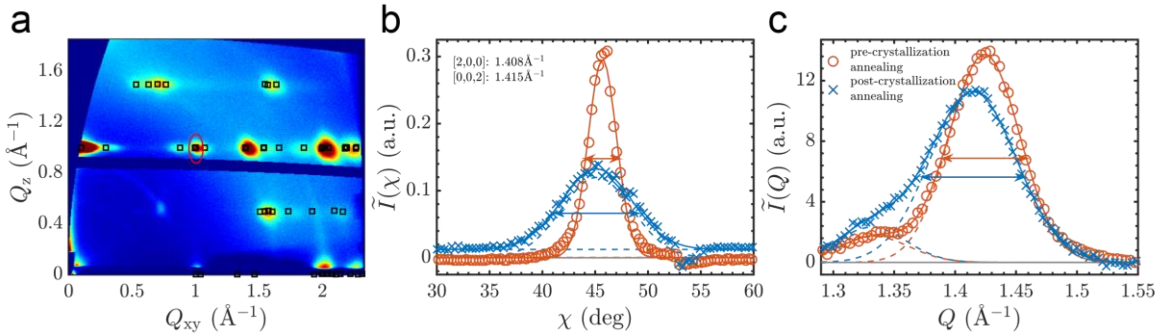

Supplementary Figure 11. 1D Gaussian fitting of the integrated intensity of Bragg peaks [2, 0, 0], [0, 0, 2] (circled in 2D GIWAXS pattern (a) of a DMAc method sample) along χ (b) and q (c) directions ($\chi = \tan^{-1}\left(\frac{Q_r}{Q_z}\right)$ is the azimuthal angle). In (b) and (c), red circle and blue cross represent $BA_2MA_3Pb_4I_{13}$ samples made by pre-crystallization annealing and post-crystallization annealing process, respectively. Peak [2, 0, 0] and [0, 0, 2] are well captured in the GIWAXS patterns and well separated from other Bragg peaks, hence chosen for the orientation analysis. These two Bragg peaks have a separation of 0.007 Å^{-1}, which is smaller than the instrument resolution (derived from the standard sample CeO_2), $\Delta Q_{res} = 0.0318$ Å^{-1}, thus can be fitted with one Gaussian function. In (b), the integrated intensity $\tilde{I}(\chi) = \int I(Q,\chi)Q\,dQ$ is taken over $Q = 1.35$ to 1.50 Å^{-1} for both samples, while in (c), $\tilde{I}(Q) = \int I(Q,\chi)\,d\chi$ is taken over $\chi = 42.0$ to 49.0 deg ($\chi = 38.0$ to 53.0 deg) for pre-crystallization annealing (post-crystallization annealing) sample. For simplicity, the data is rescaled so that the instrument background (gray solid line) is zero everywhere. The dashed line represents the single Gaussian peak and the solid line is the overall fitted curve. The double-ended arrow shows the full width at half maximum (FWHM) of the fitted Gaussian peak. The flat dashed line in (b) (with nearly no χ dependence) represents the contribution from isotropic orientated crystallites. And in (c), the small peak at 1.325 Å^{-1} comes from the reflection [1, 9, 1].

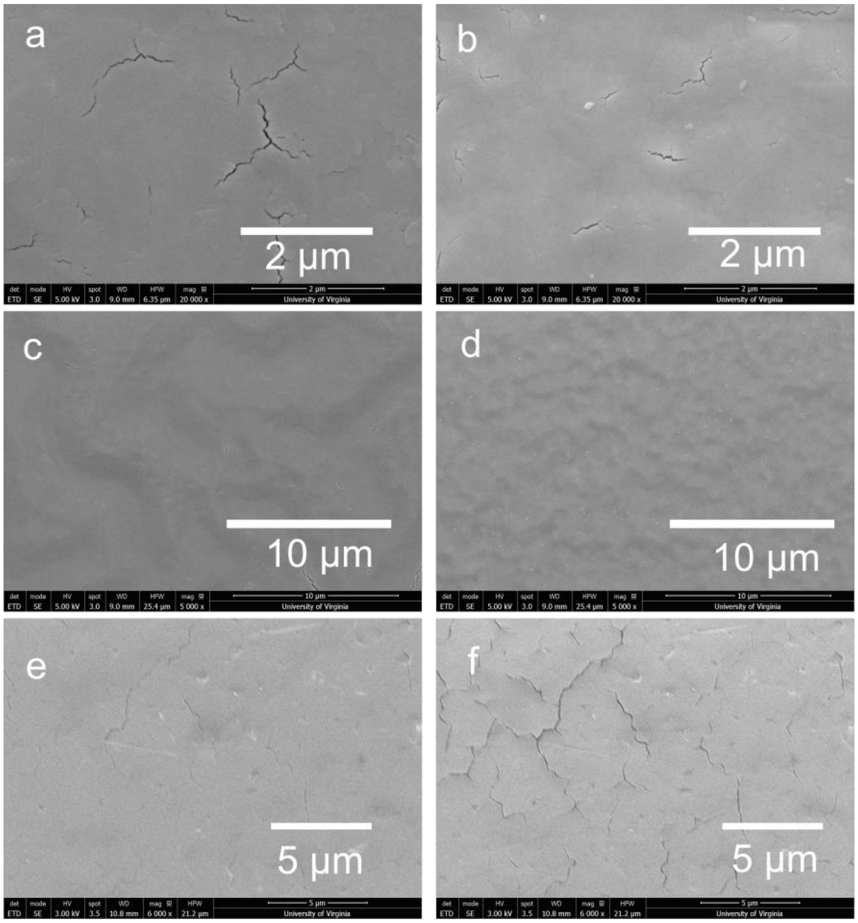

Supplementary Figure 12. SEM images of $BA_2MA_3Pb_4I_{13}$ thin films from pre-crystallization annealing (a, c) and post-crystallization annealing films (b, d). The effect of electron beam damage on the perovskite films are demonstrated by SEM of the same area after one scan (e) and 30 scans (f). This shows the cracks observed in SEM images at higher magnification are likely due to electron beam damage.

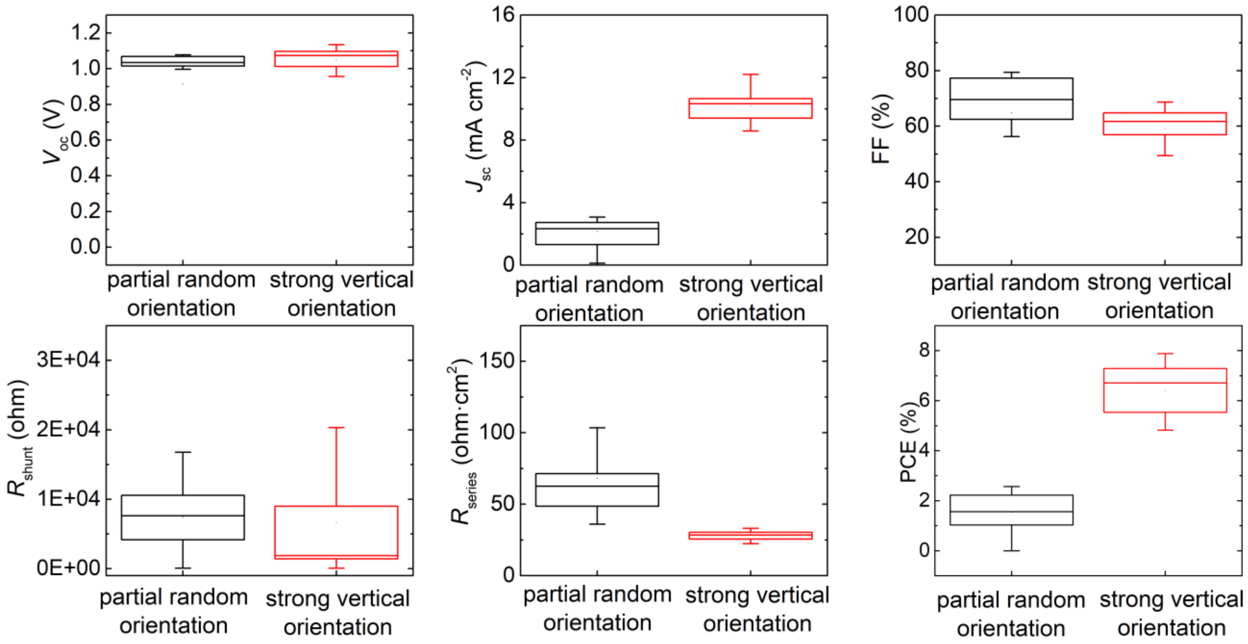

Supplementary Figure 13. Solar cell performance statistics of $BA_2MA_3Pb_4I_{13}$ devices with partial random orientation and strong vertical orientation from 30 devices for each fabrication method. Center line, 50% percentile; box, 25/75th percentiles; whisker, outlier.

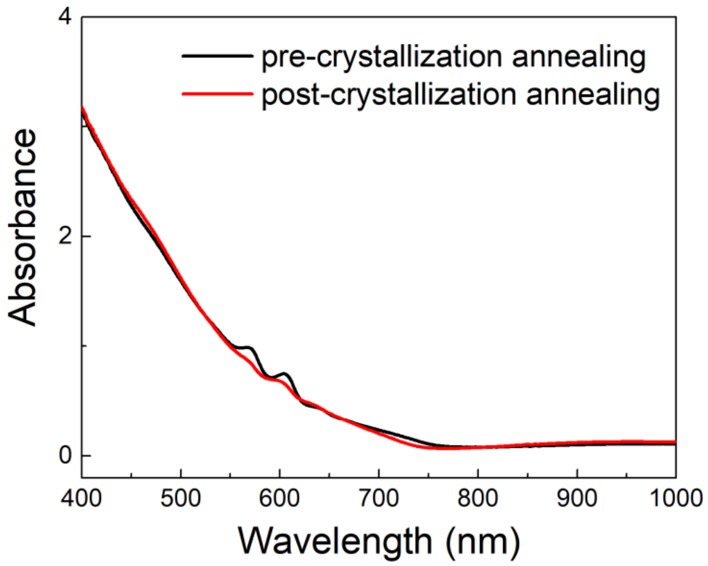

Supplementary Figure 14. Absorption spectra of $BA_2MA_3Pb_4I_{13}$ thin films from pre-crystallization annealing and post-crystallization annealing methods.

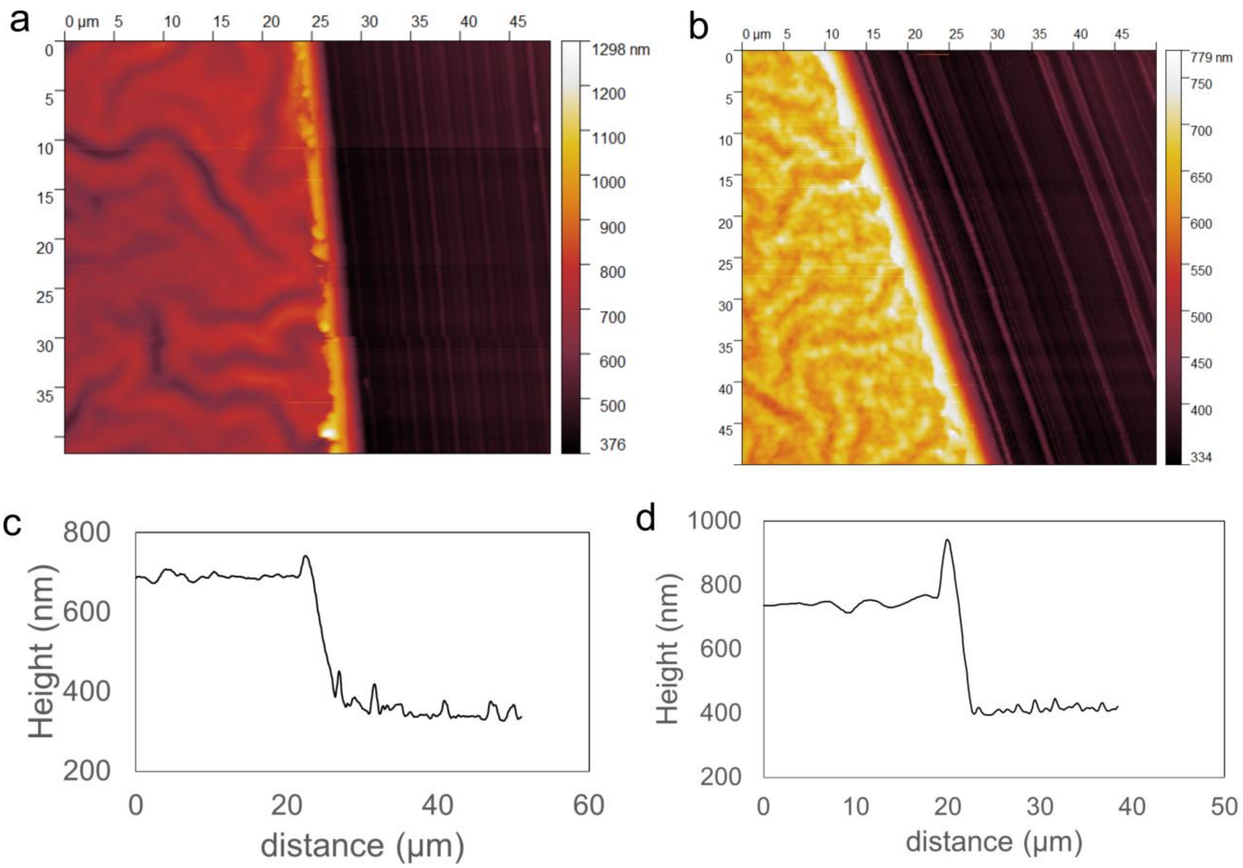

Supplementary Figure 15. Thickness measurement of pre-crystallization annealed thin film (a), (c) and post-crystallization annealed thin film (b), (d) by atomic force microscopy (AFM). The thickness was measured to be 324 nm for pre-crystallization annealed thin film and 327 nm for post-crystallization annealed thin film.

Supplementary Table 1. Fitting coefficients and orientation parameters of Bragg peaks [2, 0, 0], [0, 0, 2] calculated from the fitting results in Figure S11.

Method	Peak Center		FWHM			Correlation Length	Ratio of Orientated Crystallites
	χ_c (deg)	Q_c ($Å^{-1}$)	$\Delta\chi$ (deg)	ΔQ_{rad} ($Å^{-1}$)	ΔQ_{tan} ($Å^{-1}$)	ξ_{tan} (Å)	f_{ori}
Pre-crystallization method	45.7	1.42	3.19	0.0684	0.0792	77.9	96.0%
Post-crystallization method	45.2	1.41	7.84	0.0834	0.194	29.6	48.1%

Supplementary References

1 Zhou, H. *et al.* Photovoltaics. Interface engineering of highly efficient perovskite solar cells. *Science* **345**, 542-546, (2014).

2 Persson, I., Lyczko, K., Lundberg, D., Eriksson, L. & Placzek, A. Coordination chemistry study of hydrated and solvated lead(II) ions in solution and solid state. *Inorg Chem* **50**, 1058-1072, (2011).

3 Stoumpos, C. C. *et al.* Ruddlesden–Popper Hybrid Lead Iodide Perovskite 2D Homologous Semiconductors. *Chem Mater* **28**, 2852-2867, (2016).
